# Prospects of snap beans (*Phaseolus vulgaris L.*) production and commercialization in Benin

**DOI:** 10.3389/fnut.2023.1180134

**Published:** 2023-06-28

**Authors:** Eric Etchikinto Agoyi, Symphorien Essèdjo Ahomondji, Louis Butare, Eileen Bogweh Nchanji, Sergino Ayi, Achille Assogbadjo, Brice Augustin Sinsin

**Affiliations:** ^1^Faculty of Agricultural Sciences, University of Abomey-Calavi, Cotonou, Benin; ^2^International Centre for Tropical Agriculture (CIAT), Kigali, Rwanda; ^3^International Center for Tropical Agriculture, Nairobi, Kenya

**Keywords:** snap beans, leguminous vegetable, phaseolus, gender, production, markets, Southern Benin

## Abstract

**Background:**

Vegetables and fruits are highly recommended in diets because of their nutritional importance. Among those, leguminous vegetables are more important, for low-income countries, because of their protein, mineral contents and potential to increase food security and income. In Benin, snap beans (*Phaseolus vulgaris L.*) are the most consumed leguminous vegetables; however, their production is declining, driving the need to understand the current status of its industry to propose solutions for the revival of the sector. This paper assessed the production system, market value, marketing channels, seed systems, and the constraints associated with Benin’s snap bean production from a gender lens.

**Methods:**

A semi-structured interview was conducted with 602 bean producers and traders, randomly selected from 12 major vegetable-producing areas across Benin.

**Results:**

The study found a drastic decline in the production of snap beans, with more than 60% abandonment over the last decade. As a consequence, Benin gets supplied through importations, with the highest importation flow coming from Togo (51%), followed by Burkina Faso (25%), and Ghana (12%). Only 13% of the beans traded are locally produced. The leading causes of the decline were pests and diseases that affected the crops’ yield and quality, causing the local produce to be less valued than the imported ones. Women are heavily involved in marketing but cannot expand their business due to low production and high importation.

**Discussion:**

The study recommends that integrated pest management (IPM) and new varieties with tolerance to major pests and diseases be developed to address market demand and producers trained in agronomic practices.

## 1. Introduction

Vegetable production stimulates rural and urban economies and generates employment and income in developing countries (1). It is often carried out on small plots but results in high productivity within a relatively short period. These make vegetable production attractive, especially to young people and women. In Benin, vegetable production is more intense in southern Benin’s urban and peri-urban areas. It represents an important income-generating activity and employment opportunity for youth (2). National statistics from 2007 indicate that vegetable production has the potential to contribute to the country’s economic growth through income generation via exports to regional and international markets (3). Moreover, in the perspective of structural transformations in the national economy, the Benin Government, in its 2016–2021 Action Program and the 2025 strategic plan for agricultural sector development (PSDSA-2025), has placed the vegetable sector as one of the main vectors of wealth creation and employment (4). Vegetables and fruits are highly recommended because of their nutritional importance and numerous health benefits (5). However, due to the challenge in the cropping systems, many market gardeners need to move on to certain crops with high market and nutritional values. Among those abandoned are snap beans (*Phaseolus vulgaris L.*) (Fabales: Fabaceae), an important vegetable crop (6) grown for its tender pods and green beans. It is very rich in protein (7), essential vitamins and minerals, β-carotene (8), and fibre (9). Their consumption has numerous health benefits, including preventing cardiovascular and metabolic diseases. Nevertheless, authors reported that it is labour-intensive (10–12), thus contributing to increased employment and income generation. It is, therefore, important to work for the revival of the snap beans sector in Benin, making it necessary to discover the causes of the crop’s abandonment to design adequate solutions for that problem. The present study aims to understand the potential and constraints associated with snap beans production and commercialisation and propose solutions for the revival of the snap beans sector in Benin. Specific objectives include: (i) assessing the level of production and market value of snap beans in Benin, (ii) mapping out the supply chain of snap beans produced and seeds in Benin, and (iii) evaluating the constraints associated with the production of snap beans in Benin.

## 2. Materials and methods

### 2.1. Study area

**Figure 1:**
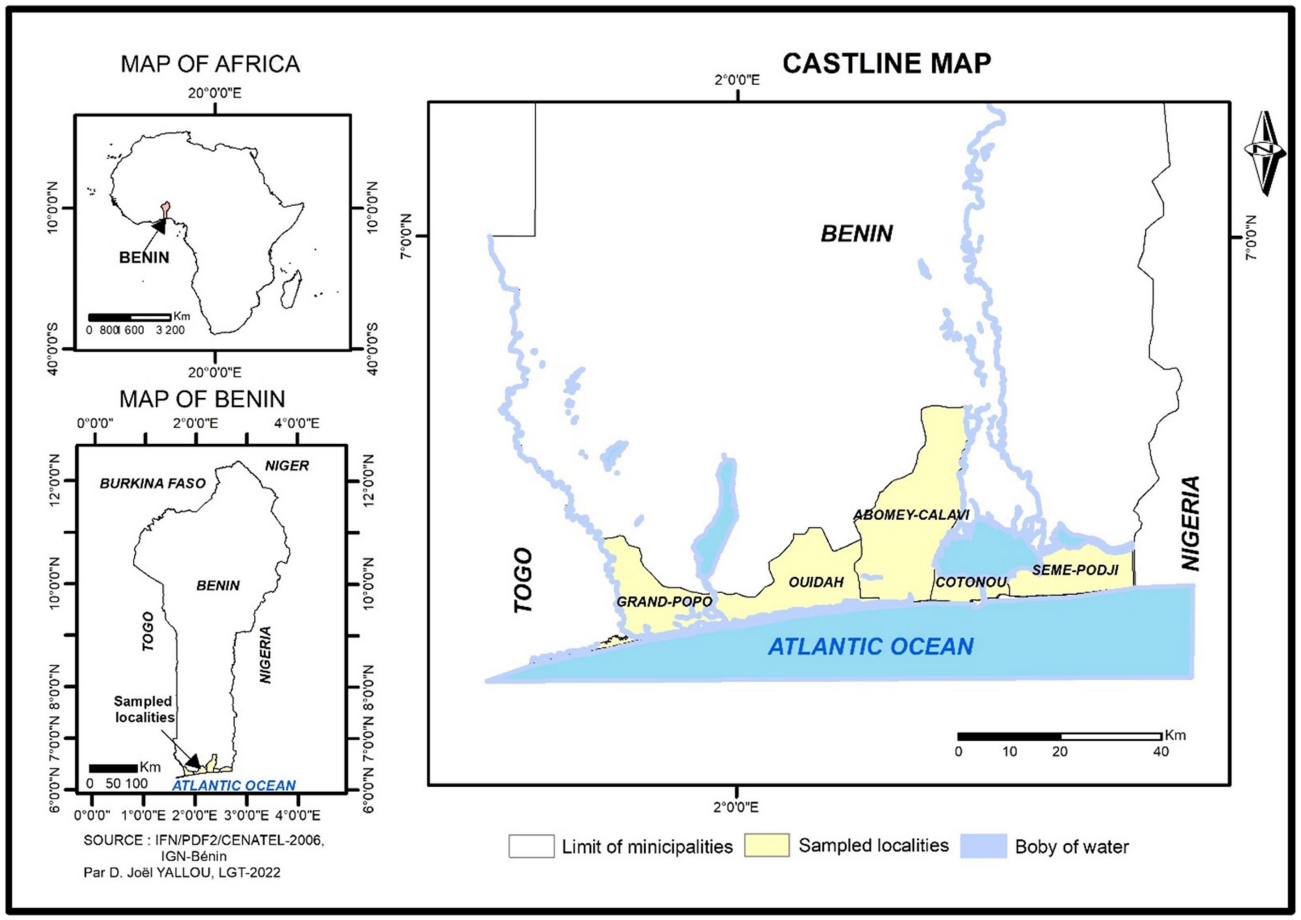
Map showing the geographical position of the five communes sampled localities.

The study was conducted in November 2022 in Benin’s four administrative departments (Atlantic, Littoral, Mono, and Ouémé). These communes represent the major agroecological zones where market gardening is well developed in Benin, and production happens. In the Department of Atlantic, Abomey-Calavi (6° 26′ 55″ N, 2° 21′ 20″E), you will find mainly tropical ferruginous and sandy soils. It is dominated by social groups such as the Aïzo and the Fon. However, the Gouns, the Nago, the Toffin, the Yoruba and others are found there. In Ouidah (6° 22′ 00″N, 2° 05′ 00″ E), there are two main types of soil – sandy and the ferralitic soil type. The population of Ouidah is composed mainly of Fon, Nago, Xuéda, and Mina. Cototou (6° 22′ 21″ N, 2° 23′ 38″ E) in the Department of Littoral is sandy and swampy. Here several ethnic groups live together. The Fon and related groups (56.5%), the Adja and associated groups (17.7%) and the Yoruba and related groups (10.9%) are the three main ethnic groups (INSAE). Grand-Popo (6° 17′ 00″ N, 1° 50′ 00″ E) in the Department of Mono consists of a sandy coastline to the south, a terminal continental shelf, and largely swampy depressions, with Xwla, Pedah and Mina as the dominant ethnic groups. The commune of Sèmè-Kpodji (6° 22′ 00″N, 2° 37′ 00″ E) in the Departement of Ouémé is sandy along the Atlantic Ocean and sandy-clay in the interior of the villages. Several ethnic groups live in the commune of Sèmè-Podji; the most dominant are the Xwla, the Goun, the Tori, the Yoruba and the Fons. A sub-equatorial climate, marked by two rainy and two dry seasons, prevails in these five communes (Figure 1).

The survey sites were chosen in consultation with the cooperative leaders of market gardeners to ensure representativeness of the past, present, and future potential snap beans production areas and facilitate access to the sites that grow the crop.

### 2.2. Sampling strategy

The sampling strategy aimed to provide the best representativeness of ethnic diversity and gender representativeness among actors in the vegetable sector. This is based on the principle that agricultural practices, beliefs and opportunities to apply technologies, may vary from one ethnic group to another and across sex and age. We selected medium-sized market sites in the communes, which were located at least 25 km apart from each other. Surveys were conducted in 12 locations across the five selected communes, using informal and semi-structured interviews and focus group discussions. Informal interviews were first conducted with the heads of the cooperatives at the sites to learn about the socio-cultural and demographic characteristics of the site regarding snap bean production and marketing. The semi-structured interviews were conducted using a questionnaire. This technique was used to explore and test their knowledge of the potentials and constraints of the snap beans sector in Benin and the role of men and women in its production and marketing. It was used to generate and classify the legume species market gardeners grew in the different targeted sites. The focus group discussions, which brought together eight to ten actors of the sector, made it possible to verify and crosscheck whether the site’s full diversity of potentials and constraints had been identified during the individual interviews. It also triangulated the data providing thick descriptions and multiple voices of the current state of affairs, production, markets, and opportunities for revamping the sector.

### 2.3. Sample size and data collection

**Figure 2:**
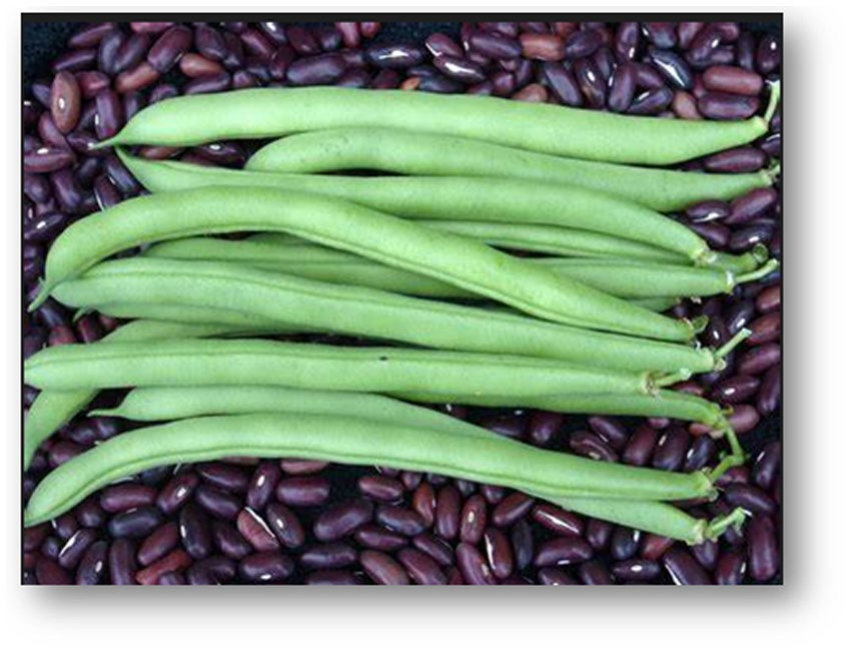
Seeds and pods of snap beans shown to informants during the interviews.

To determine the adequate sample size, preliminary surveys were conducted with 60 randomly selected individuals per location, where people were asked whether they had ever produced or sold snap beans. Pictures of snap beans seeds and pods (Figure 2) were shown to each informant.

The number *n*_i_ of people to be sampled in each region was calculated using the proportion *P* of informants who have provided positive answers, as suggested in the binomial approximation of Dagnelie (13):


$$n_{i}=U^{2}{}_{1-\alpha /2}\;\left[P\;\left(1-P\right)/d^{2}\right],$$

Where:

- *n_i_* is the total number of informants within a location;

- *U*_1−*α*/2_ = 1.96 for *α* = 0.05;

- *P* is the proportion of respondents who provided positive answers.

- *d* is the expected error margin of any parameter estimated from the interviews (*d* equals 5%).

The sum of *n_i_* from all the five selected communes gave *N* the total number of respondents sampled for the surveys.

*N* = 602 people were sampled, including 442 producers and 160 traders.

Table 2 presents the sample’s characteristics per location for the producers and their socio-cultural characteristics (age, sex, education level, ethnic group, and demographics).

The production history was investigated to document the downward trend and to highlight the degree and rate of abandonment of snap beans production in Benin. Information on production practices on snap beans and constraints was recorded. The snap beans’ marketing, supply and distribution channels were investigated with traders and other resource persons. Moreover, snap beans seeds were collected from gardeners, as shown in Figure 2 below.

### 2.4. Data analysis

Data was encoded using an Excel sheet and analysed using the R software (R version 3.6.1) (14) was used for all statistical analyses. The chi-Square test was applied to check the dependence between age groups and gender of the respondents and producers’ perception of market gardening. Moreover, a factorial correspondence analysis (FCA) was performed to describe the relationship between age groups, gender and producers’ perception of the gardening strategies. A decline curve was drawn to showcase snap beans production’s abandonment level. Descriptive statistics were used to characterise cultivation, production constraints, seed management, the origin of fresh beans on sale and the actors’ perceptions of reviving the Benin sector.

## 3. Results

### 3.1. Socio-cultural and demographic characteristics of the producers

The panel of respondents for the producers comprised 93 women and 349 men, a total of 442 people across 12 sites spread over five communes (Tables 1, 2).

**Table 1:** Number of sites and farmers surveyed per communes.

Communes	Number of sites	Total number of farmers
Abomey-Calavi	3	115
Ouidah	2	52
Grand-Popo	3	75
Cotonou	3	80
Sèmè-Kpodji	1	120
Total	12	442

**Table 2:** Characteristics of the respondents for producers.

Variables	Modalities	Size of sample	Percentage (%)
Sex	Male	349	78.96
	Female	93	21.04
	Total	442	100
Age	<25	89	20.14
	25–50	235	53.17
	50–75	104	23.53
	≥75	8	1.81
	*NA	7	1.58
	Total	442	100
School education level	None	29	6.56
	Primary	189	42.76
	Secodary	195	44.12
	University	29	6.56
	Total	442	100
Ethnic group	Adja	50	11.31
	Aïzo	68	15.38
	Fon	87	19.68
	Goun	35	7.92
	Toffin	7	1.58
	Hwla	8	1.81
	Tori	14	3.17
	Kotafon	12	2.71
	Mahi	46	10.41
	Mina	46	10.41
	Nago	29	6.56
	Pédah	18	4.07
	Sahouè	17	3.85
	Other	5	1.13
	Total	442	100

*NA, data not provided.

In all sites, fewer women were among the producers (Table 2). The age range of the interviewees was 25 to 75 years. The most dominant age group (53.17%) was middle age (between 25–50 years), and very few producers were 75 years old and above. Among those interviewees, the most representative ethnic group was the Fon (19.68%), and the least representative was Toffin (1.58%). Regarding the education level of the respondents, 44.12% of the respondents have reached secondary school, 42.76% have a primary education level, 6.56% have a higher education level, and 6.56% have never gone to school (Table 2).

### 3.2. Producers’ perception of market gardening

**Figure 3:**
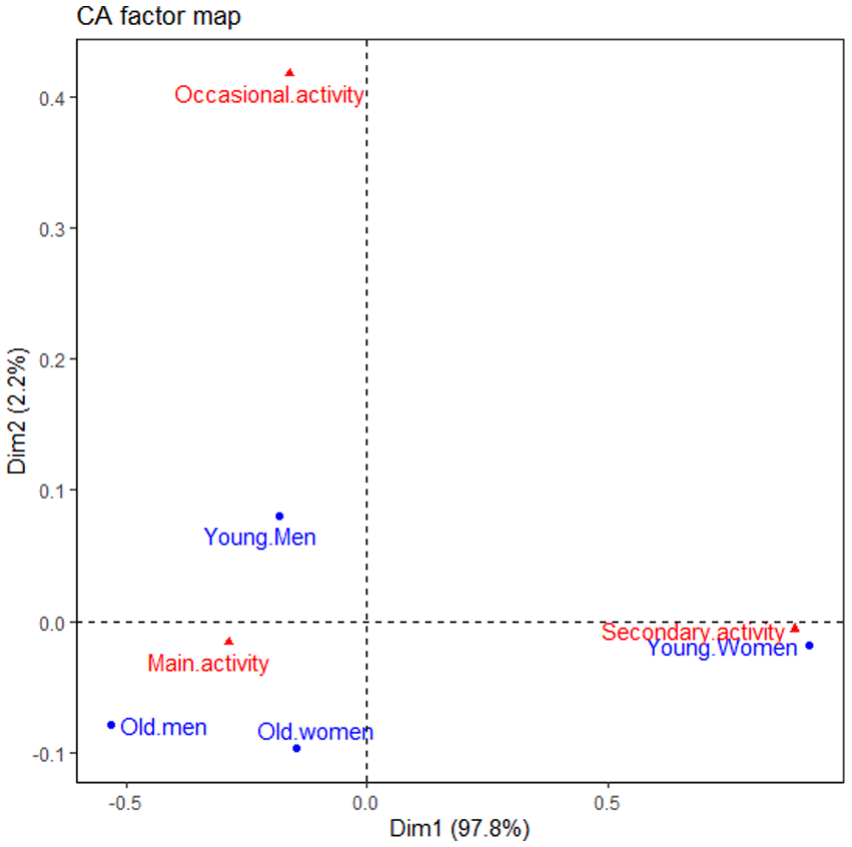
Axis system of the factorial correspondence analysis (FCA) showing the link between age, gender and market gardening activity. Blue text indicates the gender and market while the red text shows the main market gardening activity.

In Figure 3, producers’ perceptions of market gardening varied with age groups and gender (X-squared = 191.22, df = 6, value of *p* <0.001). The results of the FCA conducted to describe relationships between age, gender and producers’ perception of market gardening showed that adults (men and women) have similar prioritisation of market gardening. The prioritisation of market gardening needs to be differentiated between men and women. The contrast is proper for young people. The prioritisation is highly differentiated, with young men doing it occasionally, while young women consider it a secondary activity. While young women prioritise market gardening as a secondary activity, young men appear to be diverse in prioritising market gardening.

It is also interesting to note that young graduates and people with strong manpower who have not studied for a long time migrate to the big cities in the quest for money. For the graduates, it is mainly because of the universities concentrated in the big cities. And when they finish their university studies but do not return to the villages, they also find small jobs to earn money. As for those who need to be educated, they go to the big cities to drive the motorcycle cab, commonly called Zemidjan in Benin. According to them, climate change (floods, drought spells during the growing season) discourages their efforts in agriculture.

### 3.3. Diversity of species under vegetable cropping in Benin

Across the survey sites, snap beans had the least acreage among the vegetable crops or groups of crops grown (Table 3). Snap beans were cited by only 1.8% of the respondents, while Carrot (*Daucuscarota*) (78.1%), lettuce (*Lactuca sativa*) (78.1%), and Cucumber (*Cucumissativus*) (78.1%) were the most cited followed by leafy vegetables (68.3).

**Table 3:** Main vegetable crops grown in Benin.

,	Names	Scientifics names	Number of citations	%
01	Snap Beans	*Phaseolus vulgaris (L.)*	28	1.8
02	Carrot	*Daucus carota*	345	78.1
03	Onion	*Allium cepa*	258	58.4
04	Cabbage	*Brassica oleracea*	158	35.7
05	Lettuce	*Lactuca sativa*	345	78.1
06	Cucumber	*Cucumis sativus*	345	78.1
08	Hot pepper	*Capsicum annuum*	254	57.5
09	Beetroot	*Beta vulgaris*	128	29.0
10	Leafy vegetables (amaranth, vernonia, grande morelle)	*Amaranthus, vernonia amygdalina, Solanum marcocarpon L.*	302	68.3
10	Spices (Capsicum, ginger, garlic)	*Capsicum, Zingiber officinale, Allium sativum*	96	21.7
11	Others (Sweet corn, Potato)	*Zea mays subsp., Ipomoea batatas*	32	2.7

### 3.4. Evolutionary trend of snap beans production in Benin

**Figure 4:**
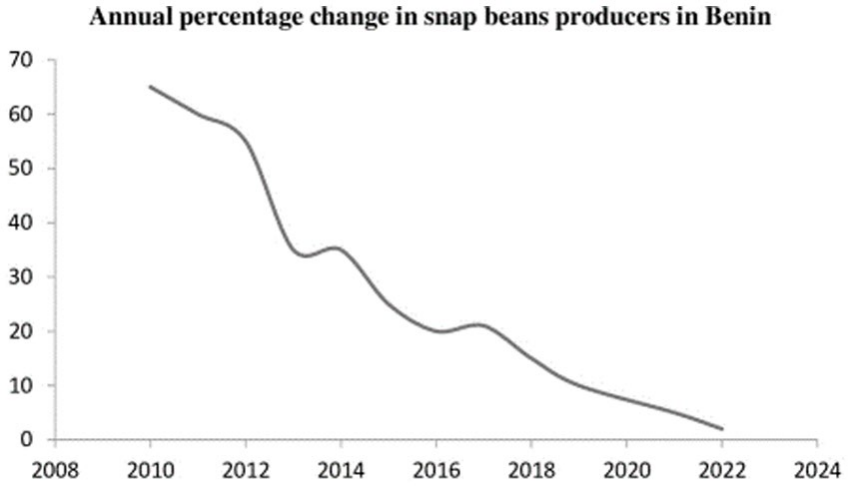
Annual percentage change in snap bean producers in Benin.

From one year to another, there is a change in the percentage of respondents that produce snap beans. From 2010 to 2022, the number of snap beans producers dropped from 65% to about 1.8%, indicating a drastic abandonment of snap beans production (Figure 4).

### 3.5. Snap beans cropping systems in Benin

**Figure 5:**
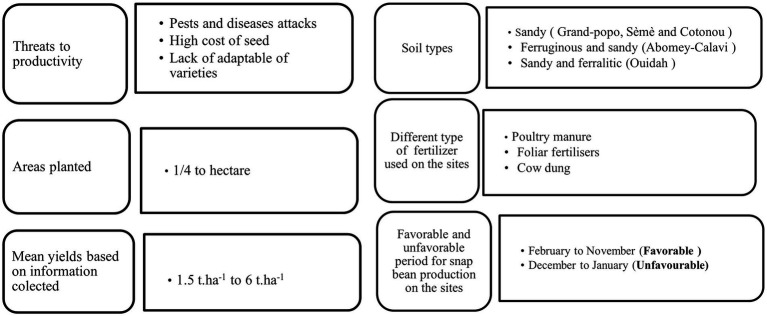
Summary of the snap bean production system in Benin.

Vegetable growers in Benin used to grow snap beans on types of soil depending on the site. The soil types in the survey areas ranged from sandy in Grand-popo, Sèmè, and Cotonou, to ferruginous and sandy in Abomey-Calavi, through sandy and ferralitic in Ouidah. The crop is often fertilised with poultry manure or cow dung and mineral fertilisers such as NPK; some use foliar fertilisers. This study revealed that producers in Benin had been growing snap beans continuously, under irrigation, from February to November. The unfavourable period ranged from December to January when the Harmattan caused a drastic reduction in relative humidity in the atmosphere. Snap bean production was carried out by smallholders (less than a hectare) with a meagre yield (1.5 t.ha-1 to 6 t.ha-1) (Figure 5). The crop does best in a rotation system and monoculture. Several pests and pathogens attack it; its production requires constant phytosanitary treatments. Based on symptom descriptions by growers, the prevailing snap beans diseases and pests have been inventoried. These were Aphids and thrips, Pod borer, White grub, Stem weevil, Stem fly, Ash weevil, Whitefly, and leafminer for pests and Powdery mildew, Rust, Anthracnose, Leaf spot, Root rot, Bacterial blight, Bacterial brown spot, Damping Off for diseases. Unfortunately, most producers complained about the lack of proper control measures for the bioagressors.

### 3.6. Snap beans seed system

**Figure 6:**
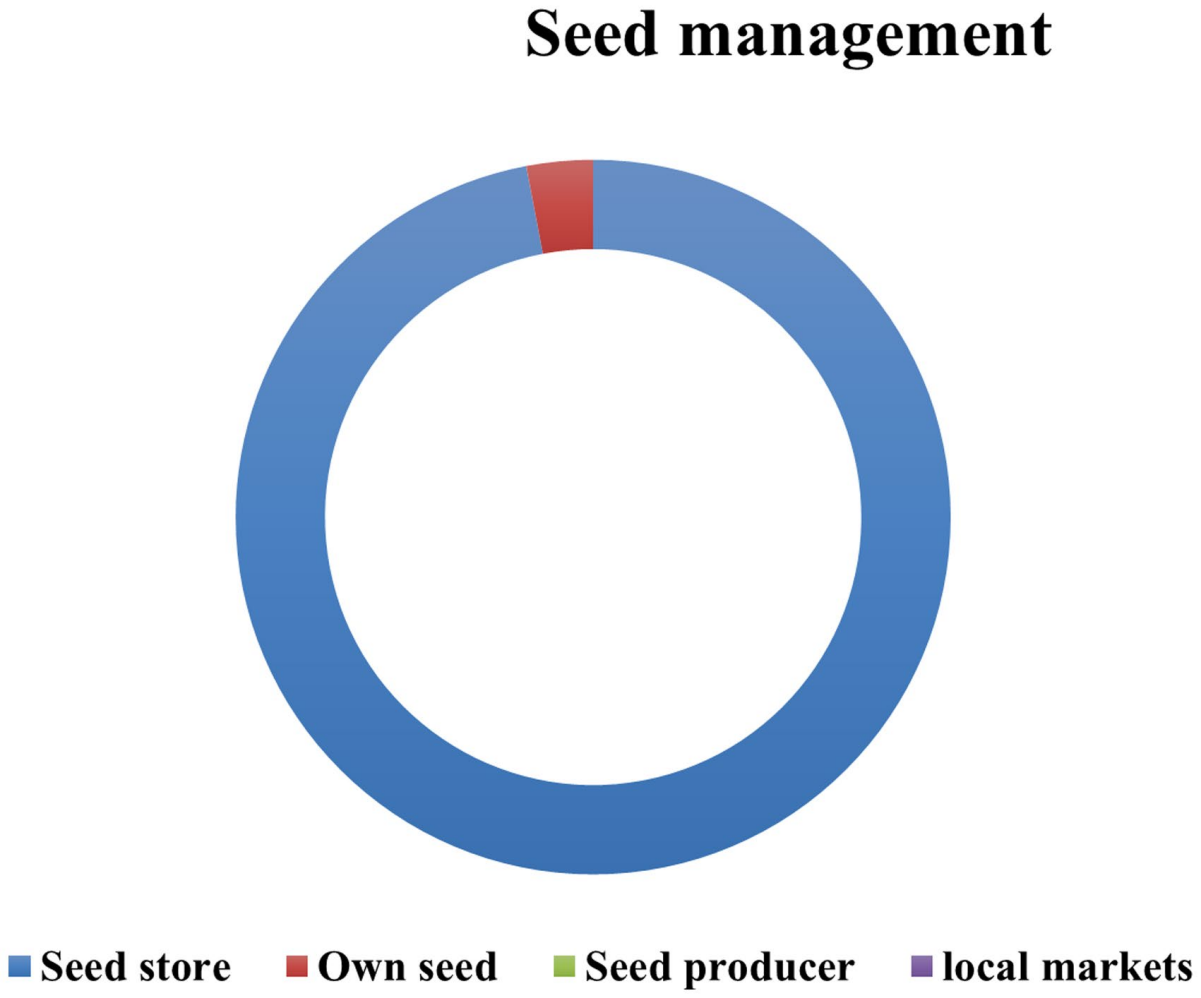
Snap beans seed management.

The prohibitive cost of seed that leads to unaffordability and lack of adaptability of varieties constitute the reasons for abandonment (Figure 5). Only one snap bean variety was collected from the sites where production continues. This variety was called ***Cora***. Most producers (97%) buy seeds of that variety from seed stores, while 3% recycle their seeds. There is no producer of snap bean seeds in Benin; all seeds come from importation, rendering snap bean seeds unaffordable (Figure 6).

### 3.7. Production constraints for snap beans

**Figure 7:**
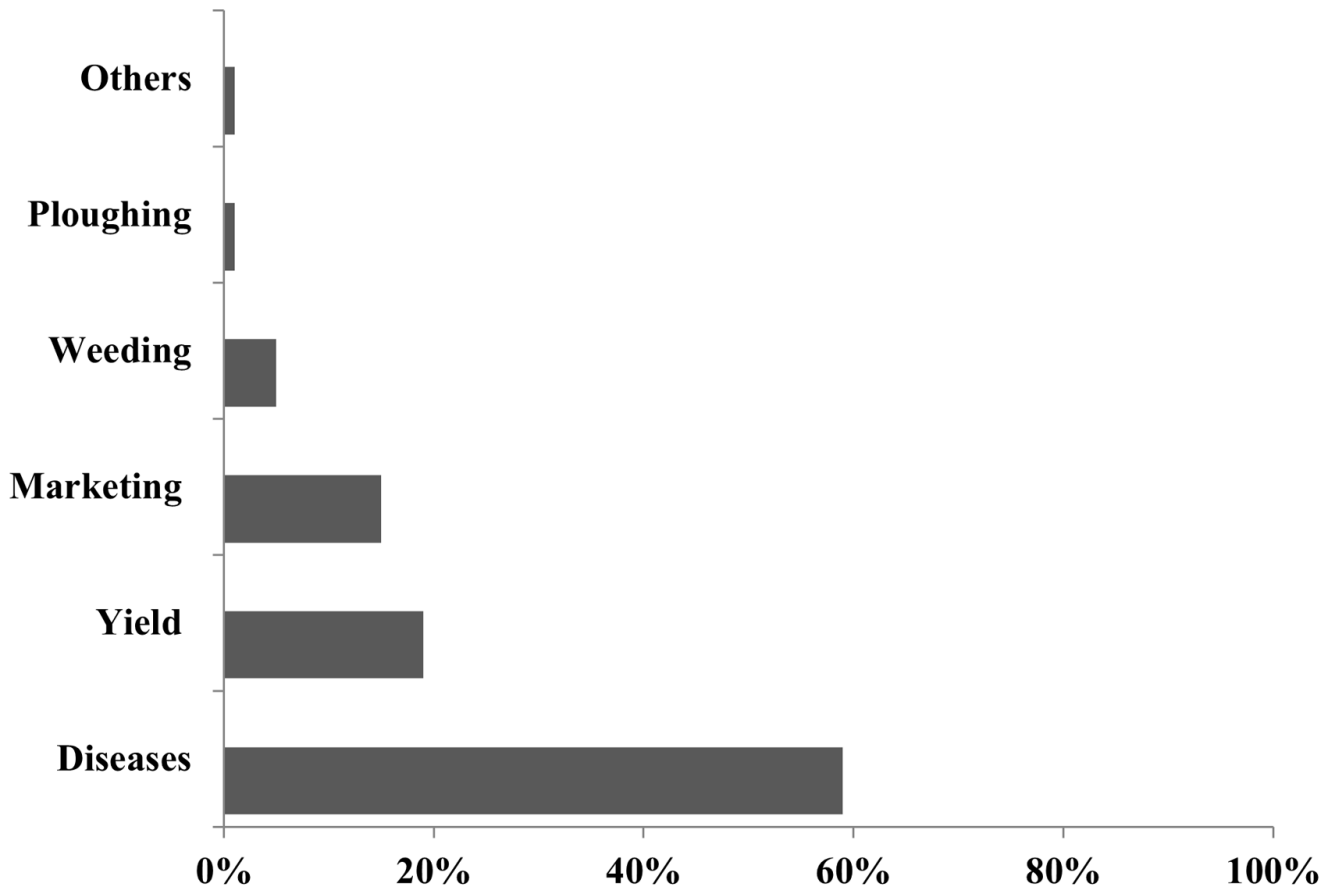
Production constraints of snap beans.

Among the constraints associated with snap beans production, pests and diseases were the most frequently cited (59%), followed by low yield (19%) and poor marketing (18%). Challenges associated with weed management (5%) and difficult ploughing (1%) were less important for producers in Benin (Figure 7).

### 3.8. Marketing of snap beans in Benin

Surveys with 162 traders enabled the identification of the supply sources of snap beans to the Benin market and importation flow. All the snap beans traders met were women aged between 35 and 65 years old. In all sites, there were only female traders of fresh legumes (Table 4).

**Table 4:** Number of sites and traders surveyed per communes.

Communes	Number of sites	Total number of traders
Abomey-Calavi	3	32
Ouidah	2	25
Grand-Popo	3	28
Cotonou	3	42
Sèmè-Kpodji	1	35
Total	12	162

**Figure 8:**
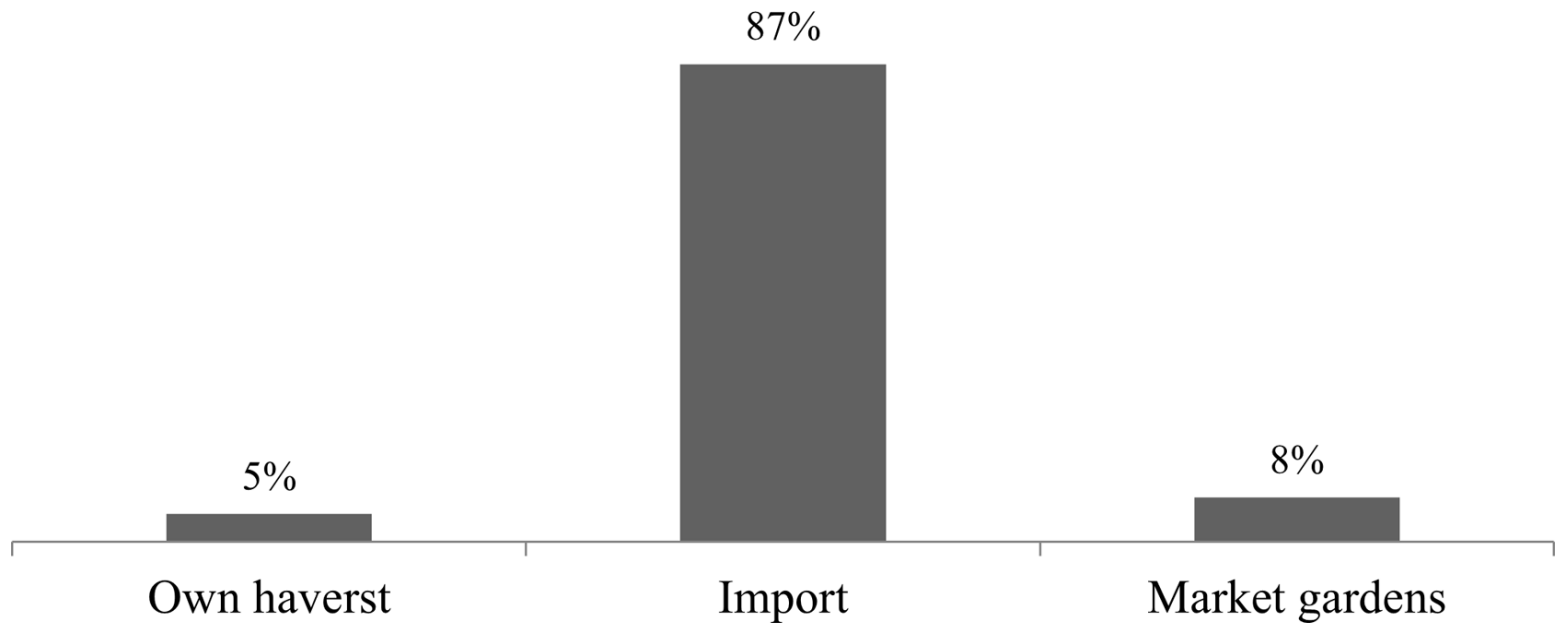
Origin of the fresh beans on sale in Benin.

**Figure 9:**
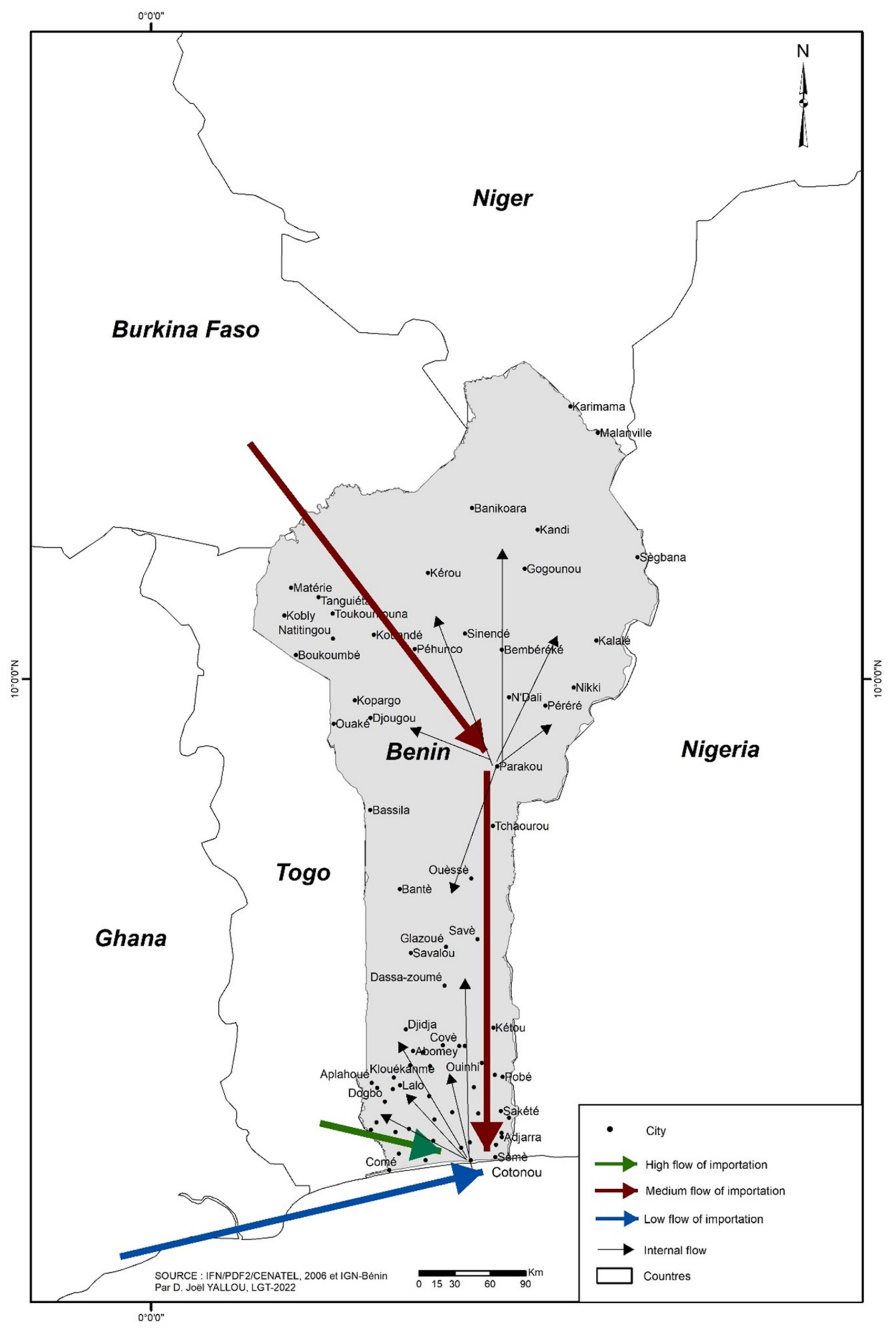
Circuit of commercialization of snap beans in Benin.

Based on respondents, 87% of the snap beans sold in Benin come from importation. Three main international sources of supply exist (Figure 8). High flow from Togo (51%), medium from Burkina Faso (25%), and low from Ghana (12%) (Figure 9). Besides, 5% of the traders get their fresh beans from their gardens, while 8% purchase their produce from Benin’s local gardens (Figure 8).

### 3.9. Local perceptions to revive snap beans production in Benin

**Figure 10:**
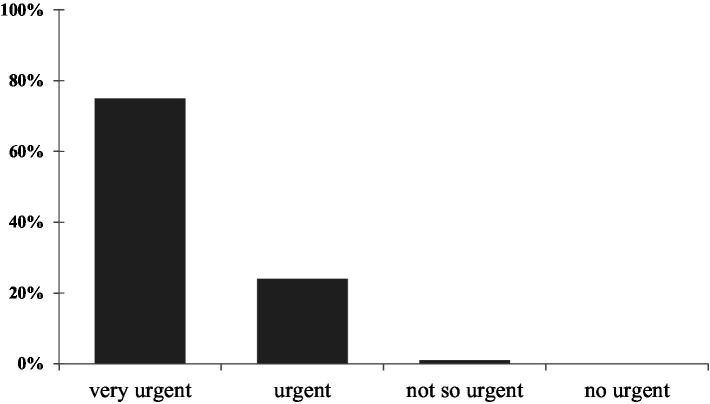
Respondents’ opinions on the need to find solutions for the revival of the sector’s production.

Most informants (65.7%) ranked the revival of the snap beans sector in Benin as a “Very urgent need” thirty point 7 % (30.7%) of the respondents ranked it as an “urgent need,” while 3.2% claimed that there is no need to revive that sector (Figure 10). This last group advocates that there is no hope for sustainable production in Benin and that the industry should continue focusing on importation rather than trying to produce locally.

**Figure 11:**
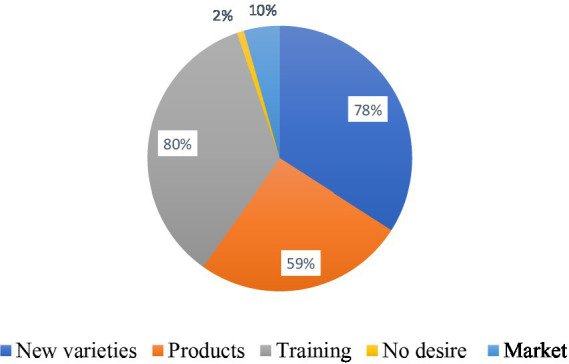
Wishes of the producers to resume the production of snap beans.

In assessing producers’ perceptions on how to revive the snap beans sector in Benin effectively, many producers (80%) expressed their willingness to resume production if high-yielding varieties with resistance/tolerance to common pests and diseases are available at affordable cost. Others (78%) perceived the availability of effective control measures for pests and diseases as an urgent action to take. Fifty-nine percent (59%) of the producers expressed the need to be trained in snap beans production, while 10% would request an improved snap beans marketing system prior to resuming production. Nevertheless, a few producers (2%) had no desire to resume production of snap beans (Figure 11).

## 4. Discussion

### 4.1. Involvement of various actors in market gardening in Benin

Based on the socio-cultural and demographic data of the study, market gardening in Benin was a viable sector that contributed significantly to reducing unemployment and addressing food insecurity in urban and peri-urban areas of southern Benin. This confirms the findings of Ouikoun et al. (15), who concluded that urban and peri-urban market gardening in Benin’s coastal cities significantly supplies households with fruits and vegetables. Moreover, most market gardeners interviewed do not have any other activities than market gardening, which explains the importance of that activity to the actors, as it generates incomes that sustain their families. This is tangible evidence supporting the Beninese government’s decisions in its action program since 2016 to develop support actions toward the market gardening actors. The majority of the vegetable growers surveyed (78.96%) were men, most of whom had high education level (Figure 4). The fact that most of the vegetable growers in the urban areas were men that have a relatively high level of education could be a result of urban migration. It is common in Benin that young men with high education migrate to town to seek a better life. Once in town, they commit to two activities: riding motor taxis and gardening. Women are few in that activity due to the patriarchal nature of the Benin population, where only a few girls are allowed to attend school. Also, precocious marriage is a plague that forces women to get married in their teenage, thus preventing them from migrating to cities or trying out activities such as market gardening. Hence, more effort must be made regarding awareness creation to encourage young women in the production sector, as only 21% are involved in the production. However, they are the sole traders of vegetables. Therefore, women are an essential link in the value chain. These observations strongly confirm those of Beune and Klaver (16), who concluded that women are increasingly included in developing the vegetable value chain in Benin. Similar results from a comparative analysis of the mango value chain in Benin, Ghana and Burkina by Van Melle and Buschmann (17). There is also a need to build men’s capacity in production to increase productivity.

### 4.2. Level of production of snap beans in Benin

Among all the vegetable species produced in Benin, snap beans were the least produced; this added to the declining trend shown in Figure 2 over the past 12 years, indicates that the crop is being abandoned by gardeners in Benin. This declined snap beans production in Benin was also reported by Ouikoun et al. (15), who, using a snowball sampling approach, did not meet any snap beans producers. On the other hand, Agoyi et al. (18) published that 52% of the respondents abandoned the production of snap. Among the 48% that still grow the crop, 17% grow it occasionally for their consumption or to meet specific demands. These results clearly show that snap beans in Benin have become a neglected crop despite their nutritional potential and market value. The present study showed that *Cora* is the only variety still in use. This contrasts with the findings of Agoyi et al. (18), who reported 8 snap beans varieties “Phenomene” climbing pole beans (52.22%), “Cora” a dwarf type of snap beans (37.36%), “hybrid” (2.42%), “variété chinoise” (1.2%), “variété hollandaise” 1.2%, “variété Japonaise” (1.2%), “kilométrique” also known as “Kaha” (1.2%) and “Monel” (1.2%), in 2019. Such a difference can be explained as the authors did not make a collection but only reported the varieties that have been or are in use at the moment of the study. However, such a big difference observed within 4 years provides further evidence of the drastic decline that the crop is experiencing in Benin.

### 4.3. Constraints related to snap beans production in Benin

The present study identified pests and diseases as the major constraints that hinder the development of the snap beans sector in Benin. A similar trend was observed by Adjatin et al. (19), who reported that insects, nematodes and pathogens, including fungi, were severe threats facing vegetable production in Benin. Agoyi et al. (18) demonstrated that in addition to pests and diseases, there need to be more good farming practices for snap beans production in Benin. Another constraint is the low yield of the varieties, discouraging many producers. In addition, Agoyi et al. (18) also indicated that consumers prefer imported products to locally produced snap beans. This raises the problem of the varieties on use that do not meet the organoleptic preferences of the consumers, but are adaptable to the environment, so they can easily be grown. Besides, the high prevalence of pests and diseases, especially the pod borers and anthracnose, alter the quality of the beans, which are likely to be rejected on the market. According to Obuobie et al. (20), the high prevalence of pests and diseases could be due to the continuous cultivation of the same species/varieties on the same plot for several years. This results in the accumulation of pathogens in the soil that significantly affects the crop in subsequent years (21). Furthermore, intensive production of irrigated vegetables (with a high number of harvests per year) can lead to nutrient depletion in soils, resulting in low yields and less resilience of weak crops regarding pest attacks (22). The issues of pests and diseases are of great concern to growers. The most severe are those that cause plant death and others that attack the pods. The detailed identification of the pests and pathogens species for each listed disease is therefore essential to design proper management approaches to boost production. Thus this information will be included in our next paper.

The production areas visited differed in terms of soil types. According to Ahomondji et al. (23), the soil pH is moderate to slightly acidic (PH < 6.5) at all of these sites, which can limit the availability of phosphorus (P), essential for nitrogen fixation. This lack of available P can be compensated using P fertilisers. In addition, except for the unfavourable harmattan period in Benin (early December to late January), when dry winds prevail, growers cultivate green beans continuously, like all other vegetable crops. In line with this, Uwiringiyimana et al. (24) found that seasons with high temperatures are unfavourable for snap bean production in Tanzania. In-depth studies must provide information on the correlation between bean cultivation, growing season, and climate (environment or physical factors) to propose adequate solutions to producers.

### 4.4. Marketing of snap beans

Despite its production chain’s drawbacks and challenges, snap beans are highly commercialised and consumed in Benin. Indeed, on the stalls of fresh vegetable sellers, snap beans are the only grain legume sold. Most of these traders obtain their supplies by importing them from neighbouring countries such as Togo, Burkina, and Ghana. This is in line with the observation made by Agoyi et al. (18), who reported Togo and Burkina Faso as the primary sources of snap beans sold in the Benin markets.

### 4.5. Perceptions of the actors on the level of the snap beans sector

The level of awareness exhibited by the stakeholders regarding the challenges and constraints of the industry and their willingness to resume if certain conditions are met indications of their readiness to work with researchers, breeders and other specialists in the agricultural sector, to implement a participatory approach toward resolution of the problems affecting the beans sector in Benin. This expressed will of the producers is an alert to researchers and policymakers at various levels who can contribute in one way or another to implementing the necessary strategies for reviving the beans sector.

## 5. Conclusion

The study pointed out that only some producers continue to invest in the production of snap beans, which used to be the only vegetable leguminous produced in Benin. The main reasons for the decline in production were pests and diseases. On the other hand, the marketing of snap beans is booming in Benin; however, most women traders get their supplies from imports. The research, therefore, needs to be pursued to avail more adapted varieties that are tolerant to bioagressors and meet market demands in terms of the quality of beans while developing and creating awareness among farmers about the technical knowledge required to achieve higher productivity and maintain quality.

## Data availability statement

The raw data supporting the conclusions of this article will be made available by the authors, without undue reservation.

## Author contributions

EA, SEA, LB, EN, SA, AA, and BS contributed to the conception and design of the study. They organized the collection of data and analysis. EA, SEA, SA, AA, and BS wrote the first draft of the manuscript. All authors contributed to the article and approved the submitted version.

## Funding

The work was supported by the Global Affairs Canada funded the research, and Bill and Melinda Gates Foundation funded the publication of this work under grant number INV-009649/OPP1198373.

## Conflict of interest

The authors declare that the research was conducted without any commercial or financial relationships that could be construed as a potential conflict of interest.

## Publisher’s note

All claims expressed in this article are solely those of the authors and do not necessarily represent those of their affiliated organizations, or those of the publisher, the editors and the reviewers. Any product that may be evaluated in this article, or claim that may be made by its manufacturer, is not guaranteed or endorsed by the publisher.
